# Phenolic Compounds in Flowers and Herb of *Achillea millefolium* L.: Histochemical and Phytochemical Studies

**DOI:** 10.3390/molecules30092084

**Published:** 2025-05-07

**Authors:** Agata Konarska, Elżbieta Weryszko-Chmielewska, Małgorzata Materska, Aneta Sulborska-Różycka, Marta Dmitruk, Barbara Chilczuk

**Affiliations:** 1 Department of Botany and Plant Physiology, University of Life Sciences, Akademicka 15, 20-950 Lublin, Poland; agata.konarska@up.lublin.pl (A.K.); elaweryszko@wp.pl (E.W.-C.); marta.dmitruk@up.lublin.pl (M.D.); 2 Department of Chemistry, Faculty of Food Science and Biotechnology, University of Life Sciences in Lublin, Akademicka 15, 20-950 Lublin, Poland; malgorzata.materska@up.lublin.pl (M.M.); barbara.chilczuk@up.lublin.pl (B.C.)

**Keywords:** yarrow, light and fluorescence microscopy, flavonoids, phenolic acids, HPLC-QTOF-MS

## Abstract

The herb and flowers of yarrow (*Achillea millefolium*) are sources of multiple bioactive secondary metabolites used in medicine and cosmetology. This study aimed to establish the location of phenolic compounds in tissues of flowers and stems of *A. millefolium* via light and fluorescence microscopy and histochemical assays. The spectrophotometric and HPLC methods were deployed to quantify total phenolic compounds (PC), phenolic acids, and flavonoids in extracts from flowers and herb, whereas the LC-QTOF-MS method was used for their qualitative analysis. The results demonstrated that PC occurred in external and internal tissues of yarrow stems, petals, and other parts of the flower, as well as in involucral bracts. Qualitative phytochemical analyses demonstrated ca. 16% more PC and flavonoids in flowers than in the herb. This analysis allowed identifying 48 PC. A higher number of PC was identified in flowers than in herbs, with rosmarinic acid followed by chlorogenic acid being the major polyphenols found in both sample types. One of the flavonoids, namely luteolin, was detected in significantly higher quantities in the flowers than in the herb. This study results provide new data on the location of PC in flowers and stems of *A. millefolium* as well as extend knowledge on their contents in the raw material of yarrow.

## 1. Introduction

Yarrow (*Achillea millefolium* L., Asteraceae) is a wild plant growing across Europe, Asia, North Africa, South Africa, and Australia [1,2]. Its aboveground parts are known sources of multiple bioactive metabolites; hence, its herb (*Millefolii herba*) and flowers (*Millefolii flos*) are used in medicine [3]. Raw materials from yarrow contain phenolic compounds, including mainly phenolic acids [4,5] and flavonoids [6,7], saponins [8], and phytosterols [9].

Phenolic acids and flavonoids are found in all plant organs; however, their contents and profiles are determined by various factors. Phenolic compounds function differently depending on the plant tissue they are located in, plant species, habitat conditions (climate, soil type), and exposure to stress induced by infections and feeding on herbivorous organisms [6,9]. Flavonoids protect plants against herbivores [10,11] and elicit protective effects under climate stress conditions [12]. In flowers, they may be found in colorful guides. They help insects find pollen, nectar, and oil, and also may serve as factors inducing pollen germination and pollen tube elongation [11]. Tannins are deterrents to herbivores owing to their toxic properties, and they form sparingly digestible complex aggregates (antifeedant) by inactivating herbivore digestive enzymes [10,13]. Changing environmental conditions have a significant impact on the distribution and content of bioactive compounds in plant organs [14,15]. For this reason, the qualitative and quantitative composition of phenolic compounds is extremely variable and cannot be explicitly determined even in the same plant species. This is why literature works often provide data on ranges of their concentrations that have been collected over the years in scientific research.

Many previous phytochemical studies have demonstrated a relationship between the concentration of phenolic compounds and the biological activity of plant extracts [5,16], including those obtained from yarrow [4]. In some cases, this activity was associated with the concentration of individual compounds. For example, Mekinić et al. [17], studying five plants including *A. millefolium*, showed that their antioxidant potential and acetylcholinesterase inhibitory activity depended on the concentration of rosmarinic acid. Similarly, Vitalini et al. [1] demonstrated that the presence of luteolin-7-*O*-glucoside and apigenin-7-*O*-glucoside determined the antiplasmodial activity of the yarrow extract.

Phenolics are deemed essential phytonutrients in health-promoting human nutrition. Regular consumption of plant-based foods containing phenolic compounds is strongly recommended due to their capability to reduce oxidative stress; minimize the risk of the development of cancers, cardiovascular diseases, and age-related neuronal degeneration; and slow down the progression of memory loss [18,19].

Phenolics are produced by different secretory structures in plants, which according to Castro and Demarco [10], can be divided into two groups: (1) cells producing mainly phenolics (epidermis, hypodermis, idioblasts and anatomical sheaths), and (2) cells producing phenolics coupled with other compounds (trichomes, cavities, ducts, laticifers, nuptial nectaries, etc.). In epidermal and hypodermal cells, phenolics are located in the vacuole. Secretory trichomes present on various plant organs differ in their structures, sizes, and locations. Their secretion is a complex mixture of different chemical compounds, including phenolic compounds [10,20,21]. The precise determination of the localization of active compounds at the tissue and cell level may be crucial in a more effective selection of specific plant parts richer in desirable compounds and enables revealing unknown aspects related to the biosynthesis, storage, and functionality of these compounds [22,23].

To date, histochemical studies focused on the localization of phenolic compounds have analyzed some species of the family Asteraceae, mainly medicinal plants. Most often, phenolic compounds in stems and leaves were determined. Their content was analyzed in various tissues [24,25,26,27,28]. The contents of phenolic compounds in different types of trichomes on leaves of several Asteraceae species were compared. These compounds were found in peltate [29] and capitate [30] trichomes. The localization of phenolic compounds was described by Bottoni et al. [31] in the secretory trichomes of *Achillea moschata* and by Asadullaeva et al. [32] in the aerial parts of *A. salicifolia*. However, there is no literature data on the distribution of phenolic compounds in the organs of *A. millefolium*.

This study aimed to provide a complex analysis of *A. millefolium* raw material via microscopic histochemical examinations and phytochemical assays. The histochemical analyses enabled the localization of phenolic compounds in the tissues of flowers and stems of the studied plant. In turn, the phytochemical assays allowed comparing the contents of total phenolics, phenolic acids, and flavonoids in its flowers and herb. Undertaking the research with *A. millefolium* was justified by the fact that its various morphotypes differ in their chemical composition [33]. Hence, it seemed advisable to investigate its various chemotypes to extend data in this area. To the best of the authors’ knowledge, there have been no studies so far that would merge the histochemical and phytochemical analyses of this species; therefore, our goal was to fill this gap. The growing demand for in-depth and explicit knowledge in the fields of phytochemistry, cosmetology, and pharmacology makes them ripe for further study.

## 2. Results

### 2.1. Histochemical Assays

**Figure 1:**
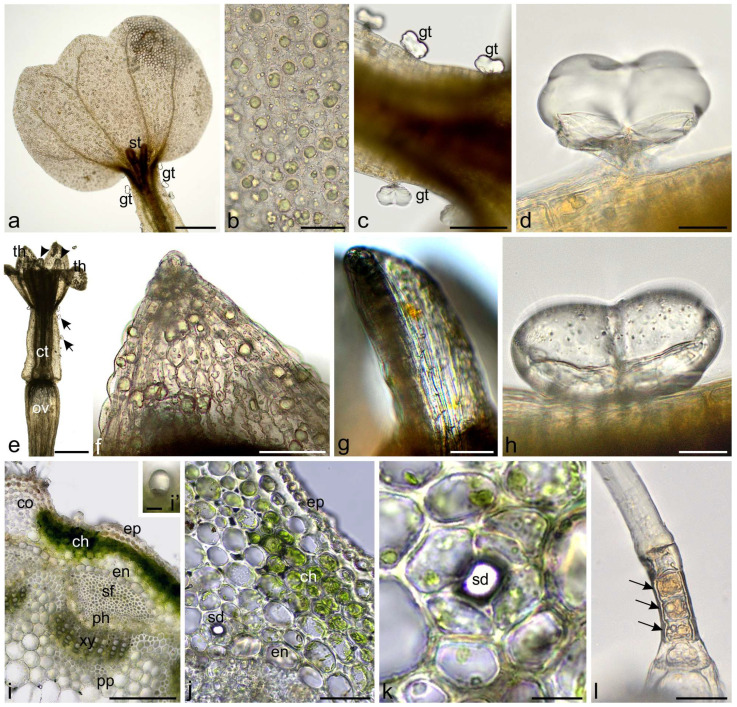
Characteristics of flowers and stems of *Achillea millefolium* (control in water, LM). (**a**–**d**) Ray floret; (**a**) Fragment of ligula and tubular part with stigma and glandular trichomes; (**b**) Epidermis of ligula with papillae; (**c**,**d**) Biseriate glandular trichomes on tubular part; (**e**–**h**) Disc floret; (**e**) General view; (**f**) Teeth of corolla with papillae; (**g**) Petal-like appendix of anther connective; (**h**) Glandular trichome on the axial surface of corolla tube; (**i**–**l**) Fragments of cross-section of yarrow stem; (**i**,**j**) Visible epidermis, collenchyma, chlorenchyma, endodermis, sclerenchyma fibers, vascular bundles, glandular trichome; (**i’**) and secretory duct (**j**); (**k**) Secretory duct; (**l**) Fragment of metabolically-active cells of the lower layer of non-glandular trichomes (arrows); st—stigma, gt—glandular trichomes, th—corolla teeth, ct—corolla tube, ov—ovary, ep—epidermis, co—collenchyma, ch—chlorenchyma, en—endodermis, sf—sclerenchyma fibers, ph—phloem, xy—xylem, pp—parenchyma pith, sd—secretory duct. Scale bars: (**a**,**e**)—500 µm, (**i**)—200 µm, (**c**,**f**)—100 µm, (**b**,**g**)—50 µm, (**i’**,**j**,**k**)—30 µm, (**d**,**h**,**l**)—20 µm.

Yarrow capitula contained female disc flowers with papillae and biseriate glandular trichomes on the corolla tube, as well as androgynous ray flowers with papillae and biseriate glandular trichomes on the corolla tube and characteristic petal-like appendices of anther connective (Figure 1a–h). Analysis of the stem anatomy revealed epidermis with disc non-glandular trichomes metabolically active in the lower part and sparse glandular trichomes, collenchyma, chlorenchyma, endodermis, clusters of sclerenchyma fibers over vascular bundles, collateral vascular bundles, and parenchyma pith (Figure 1i–l). Also, small secretory ducts were observed above vascular bundles near the endodermis (Figure 1j,k).

**Figure 2:**
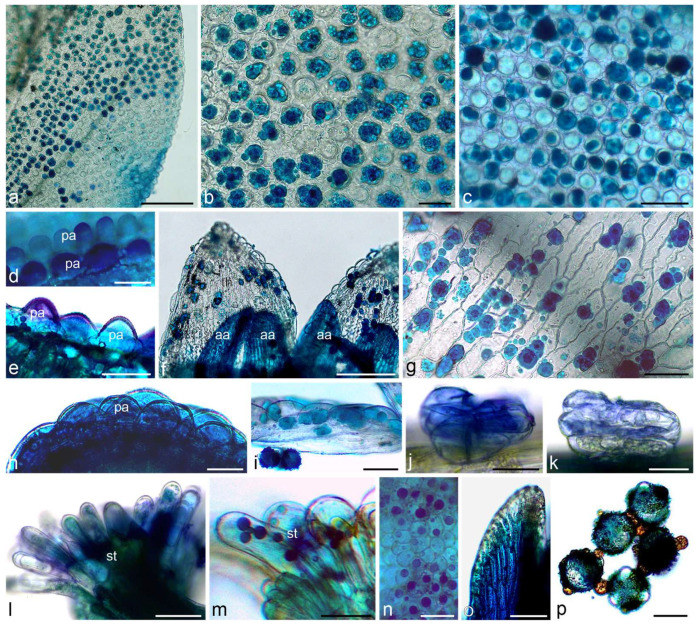
Location of total phenols in *A. millefolium* flowers. Staining with Toluidine Blue O (LM). (**a**–**e**) Turquoise and dark blue total phenols in papillae of ray floret; (**a**–**c**) Upper view; (**d**,**e**) Side view; (**f**–**i**) Total phenols in corolla tubes and appendices of anther connective of disc floret; (**f**,**g**) Upper view; (**h**,**i**) Side view; (**j**,**k**) Total phenols in glandular trichomes on ray (**j**) and disc (**k**) floret; (**l**–**n**) Total phenols in stigma papillae; (**n**) Upper view; (**o**) Total phenols in appendices of anther connective; (**p**) Total phenols in pollen grains; pa—papillae, aa—appendices of anther connective, st—stigma. Scale bars: (**a**)—200 µm, (**f**)—100 µm, (**b**–**e**,**g**,**i**,**l**,**o**)—50 µm, (**h**,**k**,**n**,**p**)—30 µm, (**j**,**m**)—20 µm.

**Figure 3:**
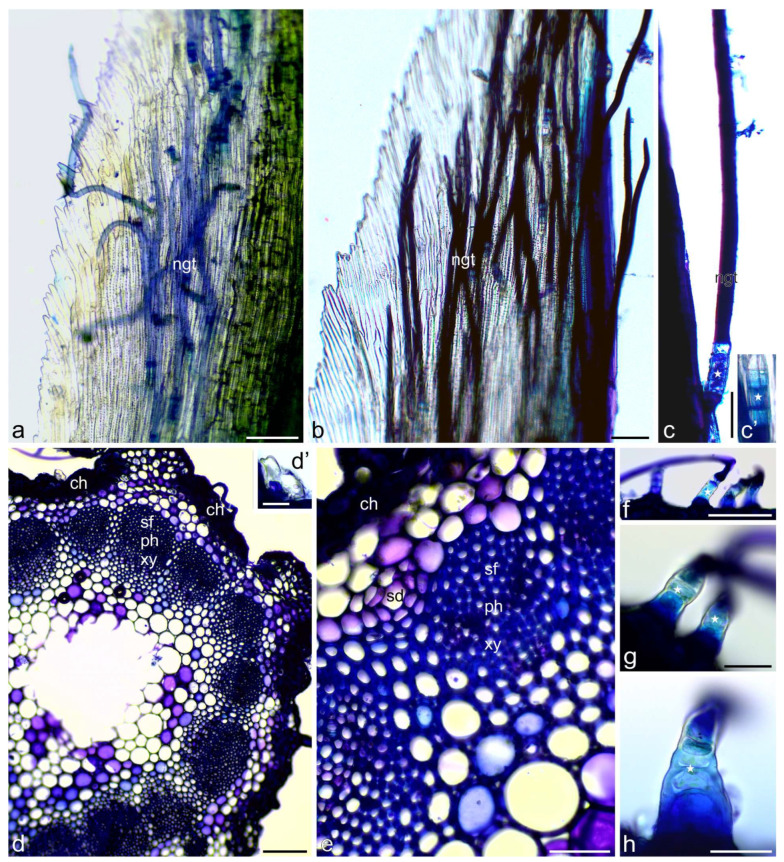
Location of total phenols in *Achillea millefolium* involucral bracts and stems. Staining with Toluidine Blue O (LM). (**a**–**c’**) Involucral bract. Dark, blue-stained total phenols present in non-glandular trichomes. Note: stained, metabolically-active cells of the lower tier of the trichomes (stars) (**c**,**c’**); (**d**–**h**) Stem; (**d**,**e**) Total phenols present in cells of epidermis and chlorenchyma, in sclerenchyma cells, xylem and phloem elements, and in glandular trichomes (**d’**); (**f**–**h**) Total phenols present in metabolically-active cells of the lower tier of the trichomes (stars); ngt—non-glandular trichomes, ch—chlorenchyma, sf—sclerenchyma fibers, ph—phloem, xy—xylem, sd—secretory duct. Scale bars: (**d**)—200 µm, (**a**,**b**,**f**)—100 µm, (**c**,**e**)—50 µm, (**d’**,**g**,**h**)—30 µm.

**Figure 4:**
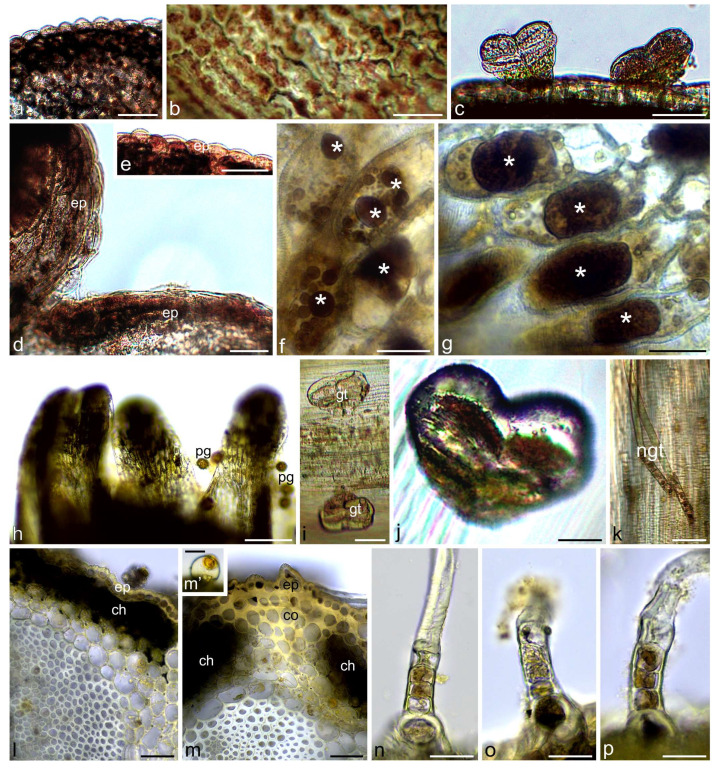
Location of total phenols in *A. millefolium* flowers and stems. Staining with ferric chloride (LM). (**a**–**c**) Ray floret. Brown-colored total phenols present in epidermal cells of ligula (**a**,**b**) and glandular trichomes (**c**); (**d**–**g**) Disc floret; (**d**,**e**) Total phenols present in epidermal cells of corolla; (**f**,**g**) Visible dark brown globules of phenols in cell vacuoles (asterisks); (**h**) Total phenols present in petal-like appendices of anther connective and pollen grains; (**i**–**k**) Bract; (**i**,**j**) Total phenols visible in glandular trichomes; (**k**) Total phenols present in cells of the lower layer of non-glandular trichomes; (**l**–**p**) Stem. Dark, brown-stained phenols present in epidermis and chlorenchyma (**l**,**m**) as well as in glandular trichome (**m’**) and cells of the lower layer of non-glandular trichomes (**n**–**p**); ep—epidermis, pg—pollen grains, gt—glandular trichomes, ngt—non-glandular trichomes, ch—chlorenchyma, co—collenchyma. Scale bars: (**h**)—100 µm, (**c**–**e**,**i**,**l**,**m**)—50 µm, (**a**,**b**,**k**,**m’**,**n**)—30 µm, (**f**,**g**,**j**,**o**,**p**)—20 µm.

Total phenols were identified in yarrow flowers and stems using Toluidine Blue O and ferric chloride. They were detected in both types of flowers and involucral bracts as well as in inflorescence stems (Figure 2, Figure 3 and Figure 4). Papillae and other cells of the epidermis of the ray and disc flower corolla contained phenolic compounds often in the form of globules of various sizes, stained in turquoise color using Toluidine Blue O (Figure 2a–i) and in brown color using ferric chloride (Figure 4a,b,d–g). Fine globules of phenolic compounds were also visible in cells of pistil stigma and style (Figure 2l–n), petal-like appendices of anther connective (Figure 2f,o and Figure 4h), and pollen grains (Figure 2p and Figure 4h) of ray flowers. They were also present in glandular trichomes of both types of flowers (Figure 2j,k and Figure 4c) and involucral bracts (Figure 4i,j) as well as in non-glandular trichomes on the bract surface (Figure 3a–c’ and Figure 4k). In yarrow stems, phenolic compounds were detected in cells of epidermis and chlorenchyma (Figure 3d,e and Figure 4l,m), in walls of sclerenchyma fibers and elements of xylem and phloem (Figure 3d,e), as well as in glandular (Figure 3d’ and Figure 4m’) and non-glandular (Figure 3f–h and Figure 4n–p) trichomes.

**Figure 5:**
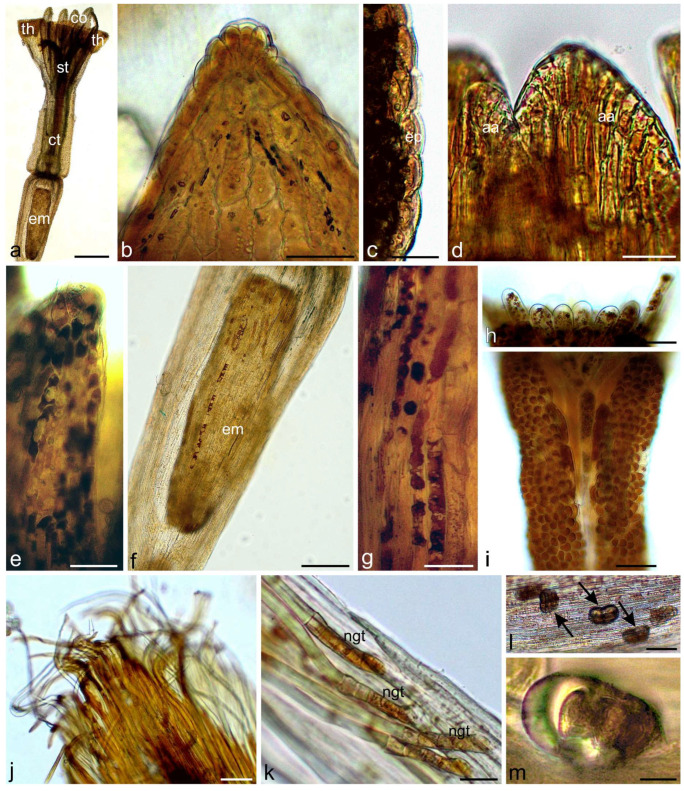
Location of tannins in *A. millefolium* flowers and involucral bracts. Staining with potassium dichromate (LM). (**a**–**i**) Disc florets; (**a**) General view; (**b**,**c**) Brown-stained tannins present in epidermis of corolla teeth; (**d**,**e**) Tannins present in appendices of anther connective; (**f**,**g**) Tannins present in embryo cells; (**h**,**i**) Tannins present in stigma papillae; (**j**–**m**) Involucral bracts; (**j**,**k**) Tannins present in epidermis cells (**j**) and cells of the lower layer of non-glandular trichomes (**k**); (**l**,**m**) Tannins present in glandular trichomes (arrows); th—corolla teeth, aa—appendices of anther connective, st—stigma, ct—corolla tube, em—embryo, ep—epidermis, ngt—non-glandular trichomes, co—collenchyma. Scale bars: (**a**)—500 µm, (**f**)—200 µm, (**j**,**l**)—100 µm, (**b**–**e**,**g**,**i**,**j**,**m**)—50 µm, (**h**,**k**)—30 µm.

**Figure 6:**
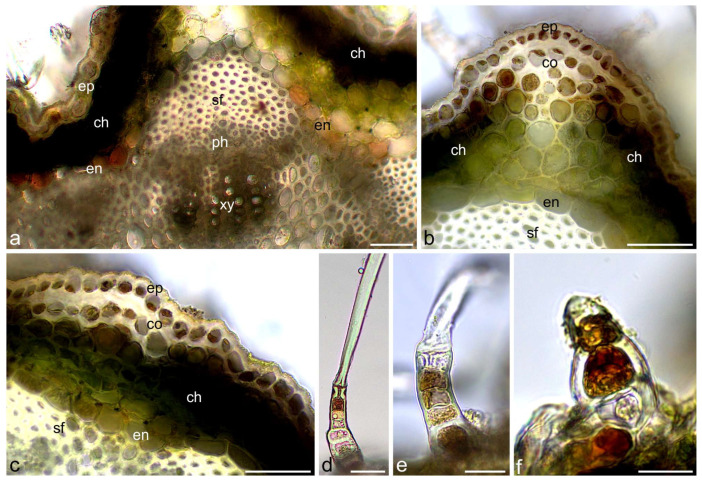
Location of tannins in *A. millefolium* stems. Staining with potassium dichromate (LM). (**a**–**c**) Brown-stained tannins present in cells of epidermis, collenchyma, chlorenchyma, and endodermis; (**d**–**f**) Tannins present in cells of the lower layer of non-glandular trichomes. ep—epidermis, sf—sclerenchyma fibers, ph—phloem, xy—xylem, en—endodermis, ch—chlorenchyma, co—collenchyma. Scale bars: (**a**–**d**)—50 µm, (**e**,**f**)—20 µm.

After staining with potassium dichromate, brown-colored tannins were observed in corolla teeth epidermis and in petal-like appendages of the anther connective of ray flowers (Figure 5a–e) as well as in embryo cells and papillae of the pistil stigma of disc and ray flowers (Figure 5f–i). They were also detected in elongated cells constituting bracts (Figure 5j), cells of the lower layer of non-glandular trichomes (Figure 5k), and some of the glandular trichomes (Figure 5l,m) present on the bract surface. In stems, tannins were located in cells of epidermis, collenchyma, chlorenchyma, and endodermis (Figure 6a–c), as well as in the cells of the lower layer of non-glandular trichomes (Figure 6d–f).

**Figure 7:**
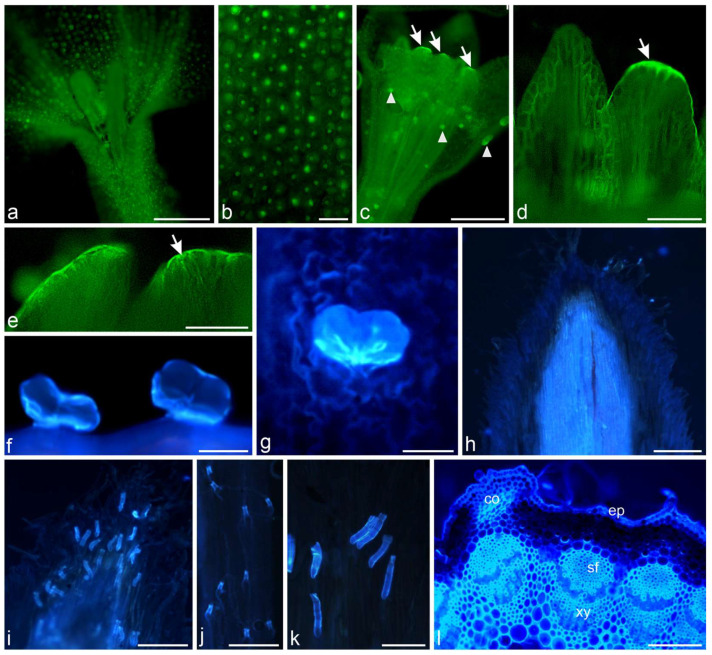
Location of phenolic acids in *A. millefolium* flowers, involucral bracts, and stems. Autofluorescence (FM). (**a**–**e**) Green filter; (**f**–**l**) Blue filter; (**a**–**g**) Flowers. (**a**,**b**) Phenolic acids present in epidermal cells of corolla of ray flowers; (**c**) Phenolic acids autofluorescence visible in corolla, appendices of anther connective (arrows) and pollen grains (arrowheads) in disc florets; (**d**,**e**) Phenolic acids autofluorescence visible in appendices of anther connective (arrows); (**f**,**g**) Phenolic acids present in glandular trichomes in ray (**f**) and disc (**g**) florets; (**h**–**k**) Involucral bracts; (**h**) Phenolic acids present in epidermal cells of involucral bracts; (**i**–**k**) Autofluorescence of phenolic acids visible in cells of the lower layer of non-glandular trichomes; (**l**) Stem. Phenolic acids present in epidermis, collenchyma, sclerenchyma fibers over xylem and in phloem elements; ib—involucral bract, ep—epidermis, co—collenchyma, sf—sclerenchyma fibers, xy—xylem. Scale bars: (**a**,**l**)—1000 µm, (**c**,**h**)–**j**—300 µm, (**k**)—100 µm, (**b**, **d**–**g**)—50 µm.

**Figure 8:**
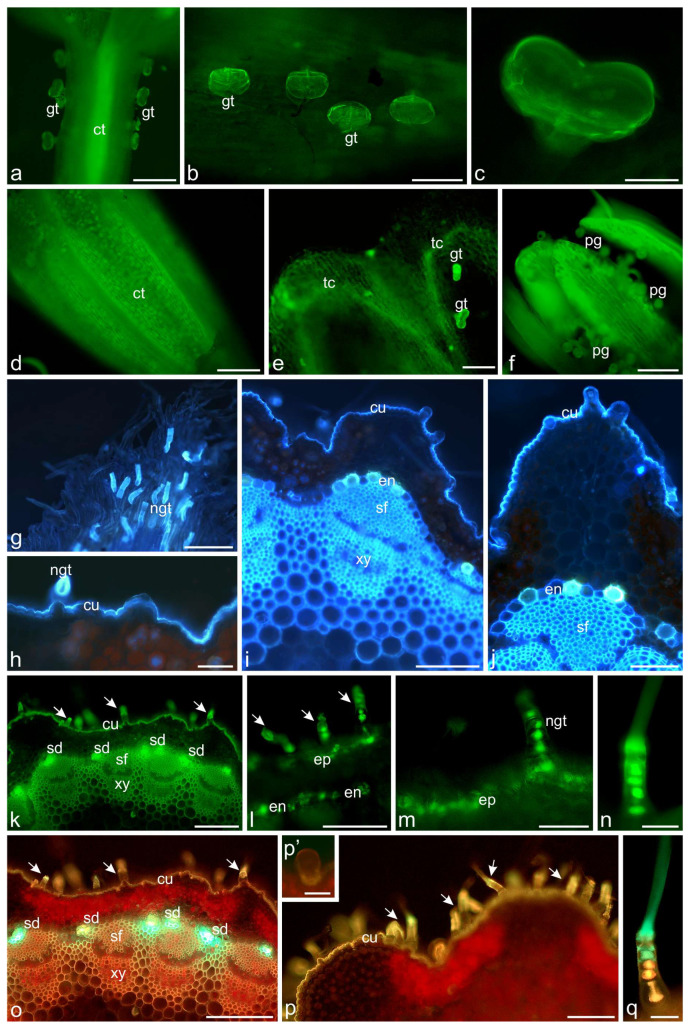
Location of flavonoids in *A. millefolium* flowers, bracts, and stems. Fluorochromes—magnesium acetate and aluminum chloride (FM). (**a**–**l**,**k**–**n**) Green filter; (**g**–**j**) Blue filter; (**o**–**q**) Green and red filters; (**a**–**c**) Ray floret. Light yellow fluorescence of flavonoids visible in the corolla tube and glandular trichomes; (**d**–**f**) Disc floret. Flavonoids in corolla tube (**d**), corolla teeth and glandular trichomes (**e**), in appendices of anther connective and pollen grains (**f**); (**g**) Involucral bract. Flavonoids present in cells of the lower layer of non-glandular trichomes; (**h**–**q**) Stem. Light-yellow fluorescence of flavonoids visible in cuticle, epidermis and endodermis cells, secretory ducts, sclerenchyma fibers and elements of phloem as well as in cells of the lower layer of non-glandular trichomes (arrows) and glandular trichome cuticle (**p’**); ct—corolla tube, gt—glandular trichomes, th—corolla teeth, pg—pollen grains, ngt—non-glandular trichomes, cu—cuticle, ep—epidermis, sd—secretory ducts, en—endodermis, sf—sclerenchyma fibers, xy—xylem. Scale bars: (**a**,**d**,**g**,**j**,**o**)—200 µm, (**b**,**e**,**f**,**i**,**j**,**l**)—100 µm, (**h**,**k**–**m**,**p**,**q**)—50 µm, (**c**,**n**,**p’**)—30 µm.

Fluorescence microscopy of both yarrow types of flowers and stems allowed identifying phenolic acids (Figure 7) and flavonoids (Figure 8).

Autofluorescence of phenolic acids was observed in corollas of disc and ray flowers (Figure 7a–c). In addition, they were located in petal-like appendages of anther connective (Figure 7c–e), pollen grains (Figure 7c glandular trichomes (Figure 7f,g), embryo cells, and stigma of pistils. Phenolic acids were also detected in cells of bracts and the lower layer of non-glandular trichomes present on the surface of bracts (Figure 7h–k). In stems, the light-blue autofluorescence of phenolic acids was noticeable in epidermis, collenchyma, sclerenchyma fibers over the xylem and phloem elements (Figure 7l).

Fluorochromes—magnesium acetate and aluminum chloride, enabled identifying flavonoids in corollas and glandular trichomes of disc and ray flowers (Figure 8a–e), as well as in petal-like appendices of the anther connective and pollen grains of ray flowers (Figure 8f). Flavonoids were also located in the cells of the lower layer of non-glandular trichomes present on the surface of bracts (Figure 8g). In stems, the light-yellow fluorescence of flavonoids was observed in cuticle, some cells of epidermis and endodermis, secretory ducts, sclerenchyma fibers, and phloem elements (Figure 8h–m,o,p), as well as in cells of the lower layer of non-glandular trichomes (Figure 8k–q).

### 2.2. Effects of Phytochemical Analyses

#### 2.2.1. Qualitative Analysis

The qualitative analysis of the extracts of yarrow herb and inflorescences was performed with the LC-QTOF-MS method (Table 1).

**Table 1:** Compounds identified in yarrow flowers and herb extracts by LC-ESI-QTOF-MS.

No.	RT	Tentative Identification	Formula	M/Z	Flowers	Herb	Refs.
	(Min)						
1	1.77	Caffeic acid 3-glucoside	C_15_H_18_O_9_	343.1021	+	+	
2	1.99	Salicylic acid	C_7_H_6_O_3_	139.0395	+	+	
3	2.71	Chlorogenic acid	C_16_H_18_O_9_	355.1027	+	+	Standard [5,34,35]
4	3.64	Myricetin 3-*O*-rutinoside	C_27_H_30_O_17_	627.1559	+	−	
5	3.69	Quercetin 3-*O*-rutinoside-7-*O*-Gluc	C_33_H_40_ O_21_	773.213	+	+	
6	3.77	*p*-Coumaroyl quinic acid	C_16_H_18_O_8_	339.1077	+	+	[5,34]
7	4.01	Kaempferol 3,7-*O*-diglucoside	C_27_H_30_O_16_	611.1618	+	+	
8	4.07	Quercetin 3-*O*-xylosyl-rutinoside	C_32_H_38_O_20_	743.2036	+	−	
9	4.09	3-Feruloylquinic acid	C_17_H_20_O_9_	369.1199	+	+	
10	4.14	Isorhamnetin 3-*O*-rutinoside	C_28_H_32_O_16_	478.1111	−	+	
11	4.21	Cyanidin 3-*O*-rutinoside	C_27_H_31_O_15_	596.1695	+	+	
12	4.25	Kaempferol 3-*O*-rutinoside	C_27_H_30_O_15_	595.1658	+	−	[5,34]
13	4.29	Apigenin7-*O*-apiosylglucoside	C_26_H_28_O_14_	565.1562	+	+	
14	4.34	Caffeic acid	C_9_H_8_O_4_	181.0507	+	+	Standard [35]
15	4.52	Quercetin 3-*O*-galactoside	C_21_H_20_O_12_	465.1052	+	+	Standard
16	4.52	Rutin	C_27_H_30_O_16_	611.1616	+	−	Standard [5,34,35]
17	4.54	Kaempferol 3-*O*-xylosyl-glucoside	C_26_H_28_O_15_	581.1513	+	+	
18	4.54	Kaempferol 3, 7-*O*-diglucoside	C_27_H_30_O_16_	611.1627	−	+	
19	4.56	Cyanidin 3, 5-*O*-diglucoside	C_27_H_31_O_16_	612.1679	−	+	
20	4.54	3, 4 Dimethoxycinnamic acid	C_11_H_12_O_4_	209.0819	+	−	
21	4.57	Apigenin 6-*C*-glucoside	C_21_H_20_O_10_	433.1135	+	+	
22	4.59	Apigenin 6,8-di-*C*-glucoside	C_27_H_30_O_15_	595.1667	+	+	[5]
23	4.63	Quercetin 3-*O*-glucoside	C_21_H_20_O_12_	465.1052	+	+	Standard [34,35]
24	4.65	Luteolin 6-*C*-glucoside	C_21_H_20_O_11_	449.1085	+	+	Standard [5]
25	4.67	Delphinidin 3-*O*-glucoside	C_21_H_21_O_12_	466.1093	−	+	
26	4.68	Quercetin 3′-*O*-glucuronide	C_21_H_18_O_13_	479.0835	−	+	
27	4.84	Quercetin 3-*O*-(6″-malonyl-Gluc)	C_24_H_22_O_15_	551.1055	+	+	
28	4.88	Cyanidin 3-*O*-galactoside	C_21_H_21_O_11_	450.1135	−	+	
29	4.92	Apigenin7-*O*-rutinoside	C_27_H_30_O_14_	579.1716	+	+	
30	4.95	Quercetin	C_15_H_10_O_7_	303.0502	+	+	Standard [35]
31	4.99	Isorhamnetin-3-*O*-glucoside	C_22_H_22_O_12_	479.1201	+	−	[5,34]
32	5.01	Apigenin-7-*O*-glucoside	C_21_H_20_O_10_	433.1135	+	−	Standard [5,34,35]
33	5.04	Luteolin 7-*O*-glucoside	C_21_H_20_O_11_	449.1085	+	+	Standard [34,35]
34	5.09	Apigenin 7-*O*-glucuronide	C_21_H_18_O_11_	447.0934	+	−	[34]
35	5.12	3,4-Dicaffeoylquinic acid	C_25_H_24_O_12_	517.1366	+	+	[34]
36	5.17	Ferulic acid	C_10_H_10_O_4_	195.0665	−	+	Standard
37	5.18	Chrysoeriol 7-*O*-glucoside	C_22_H_22_O_11_	463.1233	+	+	
38	5.21	Quercetin 3-(6″-acetylglucoside)	C_23_H_22_O_13_	507.1145	−	+	
39	5.39	Quercetin 3-*O*-rhamnoside	C_21_H_20_O_11_	449.1076	+	−	[35]
40	5.42	Isorhamnetin	C_16_H_12_O_7_	317.0658	+	−	
41	5.62	Cyanidin 3-*O*-(6″-acetylglucoside)	C_23_H_23_O_12_	492.1286	−	+	
42	5.75	Genistein	C_15_H_10_O_5_	271.0604	+	−	
43	5.86	Luteolin	C_15_H_10_O_6_	287.0552	+	+	Standard [5,34,35]
44	5.91	Naringenin 7-*O*-glucoside	C_21_H_22_O_10_	435.1277	−	+	
45	6.17	6″-*O*-Acetylgenistin	C_23_H_22_O_11_	475.124	+	−	
46	6.27	Glycitein 7-*O*-glucoside	C_22_H_22_O_10_	447.1297	+	+	
47	6.59	Apigenin	C_15_H_10_O_5_	271.0603	+	+	Standard [5,34,35]
48	6.89	Rosmarinic acid	C_18_H_16_O_8_	361.0922	+	+	Standard [16]

Ten of 48 identified compounds were represented by phenolic acids and their derivatives, whereas 38 were flavonoids and their derivatives. Thirteen compounds were identified based on the analysis of standard compounds and literature data. They were denoted with the numbers 3, 14–16, 23, 24, 30, 32, 33, 36, 43, 47, and 48 in Table 1. In the case of 26 compounds, their presence was confirmed in both analyzed anatomical parts of yarrow, whereas 10 compounds were detected only in the herb and 12 only in the flowers (Table 1).

#### 2.2.2. Quantitative Analysis

Quantitative analysis of the extracts prepared from yarrow herb and inflorescences was conducted via spectrophotometric and HPLC methods. The first served to determine the total content of phenolic compounds, which was then expressed in gallic acid equivalents, and the total content of flavonoids, later expressed in quercetin equivalents (Table 2). The contents of both total phenolics (TPC) and total flavonoids were 16% higher in the extracts from flower capitula than in those from the herb.

**Figure 9:**
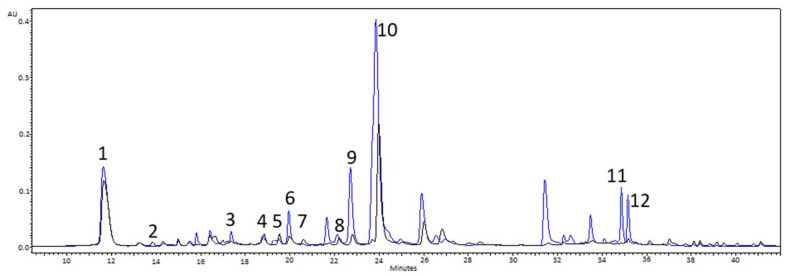
Chromatograms of flowers (blue line) and yarrow herb (black line) recorded at l = 320 nm with marked peaks of quantified compounds; 1: Chlorogenic acid; 2: Caffeic acid; 3: Rutin; 4: Luteolin-7-*O*-glucoside; 5: Quercetin 3-*O*-glucoside; 6: Ferulic acid; 7: Quercetin 3-*O*-galactoside; 8: Apigenin-7-*O*-glucoside; 9: Salicylic acid; 10: Rosmarinic acid; 11: Quercetin; 12: Luteolin.

Chromatographic analysis of yarrow inflorescences and herb demonstrated that the profile of their phenolic compounds varied, with phenolics strongly predominating in the capitula extracts. Chromatograms of yarrow herb and inflorescence extracts with numbers ascribed to the identified compounds are presented in Figure 9. The comparison of retention times and UV-Vis spectra of the acid and flavonoid standards allowed identifying twelve compounds, including five phenolic acids and seven flavonoids with their derivatives. Most of the quantified compounds prevailed in the extract from yarrow flowers, except for luteolin-7-*O*-glucoside and quercetin 3-*O*-glucoside, which predominated in the herb as well as apigenin-7-*O*-glucoside, whose content was comparable in both types of extracts (Table 2). The major acid found in both types of extracts turned out to be rosmarinic acid (no. 10 on the chromatogram) (Figure 9). Its concentration in the capitula extract was over twofold higher than in the herb extract and accounted for over 30% of the weight of all compounds quantified in the inflorescence extract (Table 2). Salicylic acid (no. 9) was the second compound in terms of concentration determined in the capitula extract (Figure 9) and accounted for almost 25% of the weight of the quantified compounds (Table 2). In the case of herb extract, the second compound in terms of concentration turned out to be chlorogenic acid, accounting for ca. 27% of the weight of the quantified compounds (Table 2). Concentrations of the seven quantified flavonoids and their derivatives were lower compared to those of the major acids (Figure 9). The major flavonoid of the flower head extract was luteolin (no. 12), accounting for over 19% of the weight of the quantified compounds (Table 2). In turn, quercetin-3-*O*-glucoside (no. 5) was the prevailing compound of the herb extract, with its content accounting for 25% of the weight of all quantified compounds (Table 2).

## 3. Discussion

### 3.1. Histochemistry

The histochemical section of this manuscript presents findings related to the location of phenolics in stems and flowers of *A. millefolium*. In studies of other researchers, some investigations have addressed the location of phenolic compounds in trichomes of Asteraceae stems. They show that these compounds were detected in glandular trichomes of *Santolina ligustica* [36], some trichomes of *Mikania cardifolia* [27], and *Achillea salicifolia* [32]. The present study demonstrated that phenolics were detected in both glandular and non-glandular trichomes of *A. millefolium* stems. They were observed in stem cells and the secretion of glandular trichomes, as well as in cells forming the lower level and apical, long cells of non-glandular trichomes.

Phenolic compounds were also detected in various tissues of *A. millefolium* stem, i.e., cells of epidermis, collenchyma, chlorenchyma, and some cells of endodermis, as well as in walls of sclerenchyma fibers and elements of xylem and phloem. In turn, flavonoids were detected in the secretory ducts of yarrow stems. In an earlier study addressing the Asteraceae family, phenolic compounds were also detected in the secretion of stem secretory ducts of *Centaurea cyanus* [37].

Results of the present study are consistent with literature data demonstrating the occurrence of phenolics in stem tissues of different species, including epidermis [26,27,32], hypodermis [28], parenchyma cells [24,32], and phloem cells [26].

In the case of epidermal cells, phenolic compounds were located in both the cuticle and the vacuole. A previous study demonstrated that the cuticle may contain phenolic acids and flavonoids, which are of physiological and ecological importance [38]. Their surface location means they are easily sensed by animals to which they are usually unattractive and even toxic. In addition, flavonoids protect plant tissues against UV radiation [39]. In parenchyma cells, phenolic compounds are located in different subcellular compartments, mainly in the vacuole and the cell wall. Most often, flavonoids and tannins are accumulated in the vacuole [40], whereas phenolic acids are in the cell wall [39].

So far, sparse research has been devoted to the location of phenolic compounds in flowers and inflorescences of the Asteraceae. They were detected in trichomes of *Santolina ligustica* capitulum [36], in trichomes from *Helianthus annuus* anthers [41], in trichomes of three *Doronicum* species [30], and in capitate trichomes on *Sigesbeckia jorullensis* bracts [42]. In the disc and ray flowers and involucral bracts of *A. millefolium* analyzed in the present study, phenolic compounds were detected in glandular trichomes as well as in non-glandular trichomes on the surface of bracts. When detected using histochemical tests, they were also visible in epidermal cells of petals of the two analyzed types of flowers. Similarly, Asadullaeva et al [32] detected phenolics in corolla cells of *Achillea salicifolia*. Bottoni et al. [31] reported that glandular trichomes present in the inflorescences of *Achillea moschata* were the main site of the synthesis of polyphenols and flavonoids.

Analyses of the cells of various anatomical parts of *A. millefolium* flowers revealed globules of various sizes containing phenolic compounds in the epidermal cells of petals of ray and disc flowers, appendages of anther connective, pollen grains, and cells of pistil stigma and styles. Castro and Demarco [10] have detected phenolic globules in the cytoplasm of parenchyma cells. Phenolic compounds were also found in the cell nucleus [43], chloroplasts, and mitochondria [44].

### 3.2. Phytochemistry

The use of high-performance liquid chromatography with time-of-flight mass spectrometry enabled in-depth qualitative analysis of yarrow extracts. The present study results point to a complex chemical composition of the analyzed extracts. Based on the mass spectra of the existing patterns and the literature data on LC-MS analysis of yarrow [5,35], 48 compounds were identified in the tested samples (Table 1). Among them, nineteen were earlier identified in yarrow, which was confirmed by appropriate references (Table 1). Apel et al. [35], who analyzed the chemical composition of three species of yarrow via LC-MS, identified 25 phenolic compounds, 17 of which were detected in *A. millefolium*. The current study confirmed the presence of 12 of these compounds. In turn, Mekinić et al. [16] compared profiles of phenolic acids and antiradical activity of extracts from *Matricaria recutita*, *Achillea millefolium*, and *Helichrysum italicum* and found rosmarinic acid to be the major phenolic acid of chamomile and immortelle extracts, as evidenced by LC-MS analysis. The results of the qualitative analysis obtained in the present study are also partly consistent with findings reported by other authors [5,34].

In turn, the results of the quantitative analysis of total phenolic acids and total flavonoids in yarrow capitula and herb differed from those published by Radušiene et al. [34]. According to these authors, yarrow leaves had higher concentrations of phenolic acids, whereas flower capitula had higher concentrations of flavonoids; the flower capitula analyzed in the present study had higher concentrations of both phenolic acids and flavonoids. These differences could be due to various methodologies adopted. Radušiene et al. [34] used results of chromatographic analysis by summing up the quantified acids and flavonoids and expressing their content in µg/g dry matter. In turn, the spectrophotometric method deployed in our study enabled determining the total content of all compounds reacting positively with the Folin–Ciocalteu reagent, which could be omitted in the chromatographic analysis. In addition, most of the previous studies have analyzed yarrow herb without any division into its morphological parts [35,45] or focused explicitly on flowers [46].

Besides the aforementioned habitat conditions, which may modify the chemical composition of plants, the differences in the compared results may also be due to the extract preparation procedure and data calculation method. For example, water [45] and a 70% aqueous ethanol solution [47] were used as solvents in previous studies, whereas in the present study, extracts were prepared using 70% methanol as a solvent, and extraction was aided with ultrasounds, like in the study by Radušiene et al. [34]. The chromatographic analysis conducted in our study confirmed earlier findings [16,17] that rosmarinic acid was the major compound of yarrow extracts (Table 2). Its concentration was implicated in the antioxidative activity and acetylcholinesterase-inhibiting capability of the yarrow extracts [17]. In turn, Apel et al. [35] found chlorogenic acid to be the predominant compound in extracts from the *Achillea millefolium* herb. It was the second, in terms of concentration, acid determined in our study in the extracts from the herb, and third in the extract from the flowers of yarrow (Table 2). A similarly high concentration of chlorogenic acid was reported in aqueous extracts from yarrow herb by Georgieva et al. [45]. By means of the HPLC-DAD analysis, they identified and quantified 10 compounds from the group of phenolic acids. Three of them were also identified in our study, i.e., chlorogenic, caffeic, and ferulic acids. In addition, the aforementioned authors identified and quantified eight compounds from the group of flavonoids, with hyperoside prevailing. In turn, Radušiene et al. [34] demonstrated differences in the chemical composition of inflorescences, leaves, and stems of plants originating from Lithuania and Turkey, pointing to the important effect of geographic origin on the content of phenolic compounds. They reported over twofold higher contents of phenolic acids in the plants growing on the north. Also, they showed luteolin to be the major flavonoid of flower capitula, which is consistent with the results of the present study. In turn, Benetis et al. [46], who analyzed differences in the chemical composition of yarrow flowers collected from 22 wild habitats in Lithuania, determined the range of concentrations of phenolic acids and reported chlorogenic acid concentration between 2.837 and 12.679 mg/g. This range fails within the values determined for this acid in our study. Rosmarinic and chlorogenic acids, belonging to the group of o-dihydroxy phenolic acids, are the major active components of yarrow, as evidenced by the present study results and literature data [16,17,35]. Rosmarinic acid is also a prevailing compound of extracts from other plants belonging to the Asteraceae family, and its predominant content was confirmed in extracts from, i.a., *Matricaria recutita* [16] and *Artemisia annua* [47]. In turn, chlorogenic acid was reported as the major compound of extracts from leaves of *Balsamita major* [48], *Achillea filipendulina* [49], *A. atrata*, *A. moschata* [35], and *A. distans* [50]. Therefore, the presence of these two acids in extracts from the mentioned plants can be deemed responsible for multiple biological properties, resulting from the chemical structure of this group of compounds.

The presence of two hydroxyl groups bound with an aromatic ring in the ortho-position in a molecule, i.e., the catechol system, contributes to their specific chemical properties, like the possibility of entering into reversible redox reactions and particularly strong chelating properties [51]. Chlorogenic acid has been proven to inhibit *Staphylococcus aureus* proliferation and affect the permeability of cell membranes [52]. For this reason, the plant extracts rich in this compound may represent a promising alternative in eradicating *S. aureus* and diseases it induces, e.g., skin infections, especially in the treatment of *Acne vulgaris* [35]. Likewise, many other compounds from the group of dicaffeoylquinic acids, such as chlorogenic acid, exhibited antioxidative, anti-carcinogenic, and antibacterial activities, which were confirmed in several previous studies [53,54]. Their results enabled the conclusion that the caffeoyl groups associated with the quinic acid fragment were crucial for these activities [55]. Today, *A. millefolium* raw materials spur great interest and recognition due to their specific profile, which contributes to their hepatoprotective, neuroprotective, and cardioprotective effects, which are mainly ascribed to the anti-inflammatory and antioxidative activities of their compounds. Ali et al. [56] elucidated the mechanisms of these actions and concluded that luteolin and chlorogenic acid were their key drivers.

The microscopic observations and histochemical tests allowed locating phenolic compounds in the stems and flowers, whereas the chemical analyses made it possible to compare the content of phenolic compounds in the herb and flowers of *A. millefolium*.

## 4. Materials and Methods

### 4.1. Characteristics of Experimental Material

The experimental material included ca. 5-year-old plants of *Achillea millefolium* L. growing on natural meadows in Krężnica Jara near Lublin (southeastern Poland) (51°15′22″ N; 22°48′25″ E). The plants grew on a sandy-clay soil with a pH of 6.5, in a sunny stand with southern exposure. The plants were collected in the growing season of 2024 at the full bloom stage (20 July–30 August 2024). Voucher specimens were deposited in the Herbarium of the Department of Botany and Plant Physiology, University of Life Sciences in Lublin, Poland.

### 4.2. Microscopic Preparation and Anatomical Analyses

The material intended for microscopic analyses was derived from 10 randomly collected plants, whose inflorescences (n = 10) and apical fragments of stems (n = 10) were sampled for analyses. Disc flowers (n = 30), ray flowers (n = 30), and involucral bracts (n = 30) were prepared from fresh capitula, whereas fragments (ca. 1 cm) of stems were cut off just underneath the inflorescence.

#### 4.2.1. Light Microscopy (LM), Histochemical Assays

Fresh disc and ray flowers and involucral bracts, as well as hand-made cross-sections of stems prepared using a razor blade, were used for observations under a light microscope. Part of the material was entrapped in water with glycerin (1:1) and served as a control. The remaining material was used for histochemical assays. Total phenols were detected using Toluidine Blue O (pH 4) [57,58] and ferric chloride [59], whereas tannins were detected by means of potassium dichromate [60]. All preparations were observed under an Olympus CX23 light microscope (Olympus, Tokyo, Japan) equipped with an Olympus EP50 digital camera (Olympus, Tokyo, Japan) and EPview software ver. 1.2. Photographic documentation was prepared.

#### 4.2.2. Fluorescence Microscopy (FM)

Disc, ray flowers, and involucral bracts prepared from fresh capitula. as well as cross-sections of stems were directly mounted into water and then examined under a Nikon 90i fluorescence microscope (Nikon, Tokyo, Japan) coupled with a digital camera (Nikon Fi1) and NIS-Elements Br 2 software. Autofluorescence of phenolic acids [61] and fluorescence of flavonoids with magnesium acetate and aluminum chloride fluorochromes [62] were observed by means of a Cy5 (blue) filter set (excitation light 590–650 nm, barrier filter wavelength 663–738 nm), FITC (green) filter set (excitation light: 465–495 nm, barrier filter wavelength: 515–555 nm), and Cy5 plus TRITC (red) filter set (excitation light: 525–565 nm, barrier filter wavelength: 555–600 nm). To avoid incorrect interpretation, each assay involved a relevant number of repetitions (n = 5). The results were compared with the control.

### 4.3. Phytochemical Analysis

#### 4.3.1. Quantitative and Qualitative Analysis of Total Phenols and Flavonoids

Phytochemical analyses were conducted on the samples of plants collected at the full blooming stage. Randomly picked inflorescences (*Millefolii flos*) (50 g of fresh mass) and ca. 10 cm apical fragments of stems with leaves (*Millefolii herba*) (50 g of fresh mass) were dried in a dryer with forced air circulation at a temp. 35 °C for 2 h to achieve a constant humidity level of 12%. Afterwards, 0.5 g portions of dried and ground capitula and herb were collected to prepare solutions (1 g/100 mL) for phytochemical analyses.

The active compounds were extracted with a 70% methanol solution by means of ultrasound-assisted extraction (10 min). Next, the mixtures were centrifuged (MPW-350R, MPW, Warsaw, Poland) at 3000× *g* for 20 min, and the supernatant was decanted, evaporated, and equilibrated to a fixed volume. The prepared extract was used for spectrophotometric and chromatographic analyses.

#### 4.3.2. Liquid Chromatography-Mass Spectrometry (LC-MS) Analysis

Qualitative analysis of yarrow herb and flower extracts was performed using an Agilent Technologies 1290 series liquid chromatograph coupled to an Agilent Technologies 6530 Q-TOF LC/MS high-resolution mass spectrometer (Agilent Technologies, Palo Alto, CA, USA). A Zorbax C-18 (1.8 µm, 2.1 mm × 50 mm) column, maintained at 40 °C, was used to this end. The mobile phase consisted of 1% acetic acid in acetonitrile (A) and water (B) in a gradient proportion of solvents in which the concentration of solvent A was changed in the range of 20–90% within 19 min. The flow rate was 0.4 mL/min, and the sample injection volume was 5 µL. Mass spectra were obtained in the mass range of 100–2000 Da with a scan time of 1.0 s operated in positive (ESI+) ionization modes, under the following parameters: capillary voltage of 3500 V; nitrogen gas temperature of 300 °C at a flow rate of 5 L/min; shield gas temperature of 300 °C at a flow rate of 8 L/min, and a nebulizer pressure of 35 psi. Data were collected using MassHunter Acquisition and MassHunter Qualitative Analysis software (Agilent Technologies, Inc., Santa Clara, CA, USA). The Personal Compound Database (PCD) and Library Software system were used to interrogate the database and library directly, where the compounds were identified using the Find Compound by Formula (FBF) algorithm. The identification of compounds in this algorithm is based on the analysis of isotope patterns (the pattern can be used as a filter for elemental composition assignment). In the case of matching the isotope pattern, the deviation (ppm) between the isotopes and the monoisotopic mass, among others, is taken into account. The contributions to the overall score were set as follows: mass score 100, isotope abundance score 60, isotope spacing score 50, and retention time 100. For compounds identified on the basis of standards, the LC-MS analyses were performed in the scan mode to confirm their retention times and in the target mode to confirm their fragmentation spectra. The remaining compounds were identified based on their isotope distribution and by their comparison with information available in dedicated databases (PubChem, ChemSpider) and literature data [5,35].

#### 4.3.3. Total Phenolic Compounds

The content of total polyphenols was determined following the methodology provided by Mujic et al. [63], by means of the colorimetric method with the Folin-Ciocalteu reagent. Its reaction with compounds having a phenol structure in the alkaline medium (pH > 10) results in the reduction of molybdenum (VI) ions to molybdenum (V) ions, leading to the formation of a blue-colored complex.

For the analysis, 0.25 mL of the extract was mixed with 2.0 mL of an aqueous Folin-Ciocalteu solution obtained by a tenfold dilution of the stock reagent. After one minute, 1.0 mL of a sodium carbonate solution with a concentration of 7.5% was added to the mixture, which was then thoroughly mixed and incubated at a room temperature of 60 min. Absorbance was measured at a wavelength of λ = 725 nm against water, which served as a blank sample. Contents of phenolic compounds were computed based on a calibration curve plotted for gallic acid and expressed in mg of gallic acid/g of sample dry matter. Detailed information on the preparation of the calibration curve and the curve equation is given in the Supplementary Materials (Table S1). Analyses were performed using a Shimadzu UV-1800 spectrophotometer (Shimadzu Corp., Kyoto, Japan).

#### 4.3.4. Total Flavonoids

The total content of flavonoids was determined using a solution of aluminum (III) chloride, forming yellow-colored complexes with flavonoids. The higher the flavonoid content in the analyzed sample, the stronger the color intensity. The analysis was carried out following the procedure described by Chang et al. [64]. To this end, 0.25 mL of the analyzed extract was mixed with 0.75 mL of ethanol (96%), 0.05 mL of a 10% aluminum chloride solution, and 0.05 mL of a 1 M sodium acetate solution. Afterwards, the mixture was diluted with distilled water to a volume of 2.5 mL and thoroughly mixed. Absorbance was measured at a wavelength of λ = 415 nm after 30-min incubation at room temperature. The content of flavonoids was determined based on a standard curve plotted for quercetin and expressed in mg of quercetin/g of sample dry matter. Information on the preparation of the calibration curve and equation is given in the Supplementary Materials (Table S1).

#### 4.3.5. High-Performance Liquid Chromatography (HPLC) Analysis

The quantitative HPLC analysis was performed using an Empower-Pro chromatograph (Waters, Milford, CT, USA), which consisted of a quaternary pump (M2998 Waters, Milford, CT, USA) with a degasser and a UV-Vis diode array detection (DAD) system (2998 Photodiode Array Detector, Waters, Milford, CT, USA). Separation was performed on a column filled with modified silica gel RP-18 (Atlantis T3—Waters, 3 µm, 4.6 mm × 150 mm). The mobile phase consisted of solvents A (1% acetic acid) and B (acetonitrile) in a proportion in which the concentration of solvent B was as follows: 8–12% until the 0–8th min–, 20% in the 10th min, and 25% in the 25th min; the flow rate was 1 mL/min. The detection was carried out at 320 nm. Compounds denoted in the chromatogram with numbers 1–12 (Figure 9) were identified and quantified based on standard curves prepared separately for each compound and expressed as mg/g sample dry matter.

### 4.4. Statistical Analysis

All analyses were carried out in four replicates, and standard deviations were calculated for each data series as an indicator of dataset scatter plots. A one-way analysis of variance (ANOVA) was used to assess differences in the content of individual components in yarrow herb and flowers. The significance of the differences between the means was determined using the least-squares means multiple-range test (Tukey’s test) with 5% error probability. Statistical comparisons were performed using STATGRAPHIC Centurion software, version XVI (Statgraphic Technologies Inc., Old Tavern Rd, The Plains, VI, USA).

## 5. Conclusions

The coupled use of microscopy analyses, histochemical assays, and analytical techniques in the present study allowed determining the distribution and concentration of phenolic compounds in the raw material of *A. millefolium*. Phenolic compounds were localized not only in the tissues found in the peripheral parts of the stem (epidermis, trichomes, collenchyma) but also in a few tissues located inside this organ. Phenolics occurred in all parts of florets, i.e., petals, stamens, pollen grains, pistils, and also in involucral bracts. Inflorescences of the analyzed plants had significantly higher contents of total phenolics and flavonoids and also showed a higher number of identified compounds, compared to the yarrow herb. Due to a high content of luteolin and its biological properties, yarrow flowers may offer a particularly valuable raw material with a high therapeutic potential. Because rosmarinic acid predominated in yarrow inflorescences and herb, followed by chlorogenic acid, they may be largely responsible for the biological properties of this plant. Our study provides new data on the chemistry of yarrow. Its findings underscore the importance of *Millefolii flos*, which may play a huge role in boosting the efficacy of yarrow-based products in medicine and cosmetics.

## Figures and Tables

**Table 2:** TPC and TF content in flowers and yarrow herb by spectrophotometric method, and the main phenolic content analyzed via quantitative HPLC-DAD method.

Analyzed Parameters	Herb	Flower
Total phenolic content (mg gallic acid/g)	194.59 ^b*^ ± 3.36	230.89 ^a^ ± 3.36
Flavonoid content (mg quercetin/g)	9.30 ^b^ ± 0.23	11.13 ^a^ ± 0.55
Major phenolic compounds (HPLC) (mg/g):		
Chlorogenic acid	5.992 ^b^ ± 0.042	7.612 ^a^ ± 0.015
2. Caffeic acid	0.045 ^b^ ± 0.001	0.087 ^a^ ± 0.001
3. Rutin	0.029 ^b^ ± 0.001	0.355 ^a^ ± 0.003
4. Luteolin-7-*O*-glucoside	0.555 ^a^ ± 0.001	0.472 ^b^ ± 0.001
5. Quercetin 3-*O*-glucoside	5.631 ^a^ ± 0.005	1.693 ^b^ ± 0.031
6. Ferulic acid	0.460 ^b^ ± 0.001	0.841 ^a^ ± 0.002
7. Quercetin 3-*O*-galactoside	0.179 ^b^ ± 0.001	0.039 ^a^ ± 0.001
8. Apigenin-7-*O*-glucoside	0.375 ^a^ ± 0.002	0.443 ^a^ ± 0.001
9. Salicylic acid	1.530 ^b^ ± 0.004	12.602 ^a^ ± 0.012
10. Rosmarinic acid	6.871 ^b^ ± 0.005	15.778 ^a^ ± 0.031
11. Quercetin	0.014 ^b^ ± 0.001	0.959 ^a^ ± 0.005
12. Luteolin	0.768 ^b^ ± 0.001	9.939 ^a^ ± 0.025
Total	22.45	50.82

The values are expressed as the mean ± SD (n = 3). According to the one-way ANOVA LSD test, means with a *p*-value lower than 0.05 were considered statistically different. Different letters in the same row indicate a significant difference between the results.

## Data Availability

The datasets used and/or analyzed during the current study are available from the corresponding author on reasonable request.

## Supplementary Materials

The following supporting information can be downloaded at: https://www.mdpi.com/article/10.3390/molecules30092084/s1, Table S1. Concentration ranges of gallic acid and quercetin standards at which calibration curves are linear in spectrophotometric analyses.
