# Supplementary material for: Phenolic Compounds in Flowers and Herb of Achillea millefolium L.: Histochemical and Phytochemical Studies
Source: Molecules. 2025 May 7;30(9):2084. doi: 10.3390/molecules30092084 (PMC12073966; doi:10.3390/molecules30092084)
Supplement: Supplementary file 1 [file molecules-30-02084-s001.zip › molecules-3477155-supplementary.pdf]

Calibration curves for spectrophotometric determinations

The calibration curves for the determination of the total phenolic compounds and the total flavonoids were prepared for gallic acid and quercetin, respectively. For this purpose, a series of dilutions were prepared from the starting solutions of the standards, obtaining concentrations from 0.01 to 1 mg/ml. Then, the determinations of the total phenolic compounds and the total flavonoids were performed according to the procedures described in the manuscript. Based on the absorbance values (y) of the tested solution concentrations (x), the calibration curves y = f(x) were plotted. The range of standard concentrations at which the curves were linear was determined based on the R^2 value, which was at least of the order of 0.995. The values of the linearity range and the equations of the curves are presented in Table S1.

Table S1. Concentration ranges of gallic acid and quercetin standards at which the calibration curves are linear in spectrophotometric analyses.

	Standard	Concentration range (µg/ml)	Linear regression equation	R^2
Total phenolics	Gallic acid	0.1-20	y=13.372x-0.062	0.998
Total flavonoids	Quercetin	1-10	y=66.733x-0.048	0.999
